# The skill qualification system for portal hypertension in Japan

**DOI:** 10.1002/deo2.74

**Published:** 2021-11-25

**Authors:** Masayuki Ohta, Naoya Murashima, Tetsuji Ohyama, Tomoharu Yoshida, Shozo Hirota, Hirofumi Kawanaka, Makoto Hashizume, Shinichi Nakamura, Fumio Chikamori, Susumu Eguchi, Takashi Tajiri, Katsutoshi Obara, Shigehiro Kokubu

**Affiliations:** ^1^ Global Oita Medical Advanced Research Center for Health Oita University Oita Japan; ^2^ Department of Gastroenterological and Pediatric Surgery Oita University Faculty of Medicine Oita Japan; ^3^ Department of Gastroenterology Mishuku Hospital Tokyo Japan; ^4^ Biostatistics Center Kurume University Fukuoka Japan; ^5^ Kitakyushu Kokura Hospital Fukuoka Japan; ^6^ Department of Radiology Konan Medical Center Hyogo Japan; ^7^ Clinical Research Institute and Department of Surgery National Hospital Organization Beppu Medical Center Oita Japan; ^8^ Kitakyushu Koga Hospital Fukuoka Japan; ^9^ Institute of Gastroenterology Department of Internal Medicine Tokyo Women's Medical University Tokyo Japan; ^10^ Department of Surgery Japanese Red Cross Kochi Hospital Kochi Japan; ^11^ Department of Surgery Nagasaki University Graduate School of Biomedical Sciences Nagasaki Japan; ^12^ Nippon Medical School Tokyo Japan; ^13^ Fukushima Preservative Service Association of Health Fukushima Japan; ^14^ Center for Liver Disease Minimal Invasive Treatment Shin‐Yurigaoka General Hospital Kanagawa Japan

**Keywords:** endoscopic treatment, interventional radiology, portal hypertension, skill qualification system, surgery

## Abstract

**Objectives:**

The diverse treatments available for portal hypertension require specialized knowledge of hemodynamics and include endoscopic treatments, interventional radiology (IVR), and surgery. The Japan Society for Portal Hypertension has developed the skill qualification system (SQS) for portal hypertension and began examination in 2014. Here, the status and validity of the judgment of the SQS examination were evaluated.

**Methods:**

From 2014 to 2020, 79 applicants were evaluated by the SQS for portal hypertension. Each unedited video submitted as a candidate procedure was evaluated by two judges, and a grade of greater than 70% for the scoring items assessed by the judges was required to pass the examination. Inter‐rater agreement of success/failure between the two judges was investigated by the AC_1_ coefficient.

**Results:**

The results of two judges differed for 11 of the 79 videos (13.9%), and five applicants (6.3%) ultimately failed the examination. The percentages of total points received by the applicants with endoscopic treatments, IVR, and surgery were 87.3%, 79.4%, and 80.8%, respectively. There were significant differences in the percentages between endoscopic treatments and IVR (*P* = 0.0015). The AC_1_ coefficients were 0.84 for the applicants overall, 0.93 for endoscopic treatments, 0.66 for IVR, and 0.72 for surgery. Similarly, there were significant differences in the AC_1_ coefficient between endoscopic treatments and IVR (*P* = 0.021).

**Conclusions:**

The SQS for portal hypertension of the Japan Society for Portal Hypertension showed high reliability for video assessments by the judges. This system may contribute to the spread and further development of safe and effective treatments for portal hypertension in Japan.

## INTRODUCTION

Laparoscopic surgery was introduced into Japan beginning in 1990, but several deaths from the techniques and some lawsuits were reported from the 1990s to the early 2000s due to the difficult and/or immature techniques.^1^, ^2^ The Japan Society for Endoscopic Surgery (JSES) developed the endoscopic surgical skill qualification system (ESSQS) to resolve these problems and improve laparoscopic techniques, and it began qualification examinations in 2004.^2^ The ESSQS uses unedited videotapes of an operator completing an entire procedure to evaluate laparoscopic techniques in a double‐blinded fashion. Now, laparoscopic procedures are included for five fields of surgery: gastrointestinal and general, pediatric, obstetrics and gynecology, urology, and orthopedic. To date, several reports have evaluated the judging systems.^1^, ^2^, ^3^, ^4^, ^5^, ^6^ A recent report also showed that anastomotic leakage in laparoscopic distal gastrectomy and low anterior resection occurred more infrequently with ESSQS‐certificated surgeons than with uncertificated surgeons.^7^

The Japanese Research Society for Portal Hypertension (whose antecedent was the Research Committee for Surgical Treatment for Portal Hypertension) was founded in 1967 to evaluate the results of surgical procedures for portal hypertension and develop safe and effective surgical procedures in Japan.^8^, ^9^ In 1980, the society developed general rules for recording endoscopic findings on esophageal varices to predict esophageal varices that could bleed easily.^10^ In addition, the society not only performed nationwide surveys but also revised the general rules to clarify the current status of treatments for esophagogastric varices in Japan and predict risky esophagogastric varices.^9^, ^11^, ^12^ The members of the society also performed a randomized clinical trial to clarify the prophylactic efficacy of portal non‐decompression surgery for esophageal varices.^13^ In 1994, the society joined with the Japanese Research Society for Sclerotherapy of Esophageal Varices and was renamed the Japan Society for Portal Hypertension (JSPH). Since then, this society has also performed a randomized clinical trial of prophylactic endoscopic injection sclerotherapy (EIS) for risky esophageal varices, revision of the general rules, and nationwide surveys of ectopic varices and portal vein thrombosis in Japan.^14^, ^15^, ^16^, ^17^

Symptoms of portal hypertension are variable, such as esophagogastric varices, hypersplenism, ascites, and hepatic encephalopathy. The treatments require specialized knowledge of the hemodynamics in portal hypertension, and the treatment methods are also diverse, including endoscopic treatments, interventional radiology (IVR), and surgical procedures. The JSPH has developed its own Skill Qualification System (SQS) according to the ESSQS of JSES and began qualification examinations in 2014. The main purposes are to spread and develop safe and effective treatments for portal hypertension in Japan and to contribute to the welfare of the people. The SQS also evaluates techniques for treating portal hypertension using unedited videotapes that include endoscopic treatments, IVR, and surgery. Herein, we report the first evaluation of the SQS for portal hypertension conducted by JSPH in which we assess its examination status and validity of the judgment.

## METHODS

### SQS and judging committees

The SQS committee, which consists of 11 executive members of the JSPH, was organized in 2010, and discussed and prepared the requirements for applicants, the review methods, the candidate procedures, and the criteria and scoring items of the examination. It spent more than 3 years establishing the SQS for portal hypertension, and the candidate procedures in the 3 specialized fields are shown in Table 1. The criteria of each procedure were divided into two categories, common and specific criteria, according to the ESSQS of JSES,^2^, ^3^, ^4^ and the examination criteria of EIS for esophageal varices using 5% ethanolamine oleate with contrast iopamidol (EOI) under fluoroscopy, balloon‐occluded retrograde transvenous obliteration (B‐RTO) and laparoscopic splenectomy are showed in Tables 2, 3, 4. The full score for each procedure was 100 points, and a score of greater than 70 points was initially considered to be required to pass the examination. Then, 29 experts in portal hypertension who were considered active members of the JSPH were recommended by the SQS committee, and the judging committee was organized. Their fields of expertise were endoscopic treatments for 12, IVR for nine, and surgery for eight experts, and each was in charge of only one field. The members of the judging committee mutually reviewed the unedited videos of entire procedures performed by other members in 2013 to confirm their technical skills and evaluate the scoring items and examination criteria. These experts were all considered to have sufficient technical skills to judge the examination. Some judges suggested that occasionally, some scoring items could not be assessed. Therefore, a grade of greater than 70% for the scoring items assessed by the judges had become the criterion of success. The names of the judges had been kept confidential.

**TABLE 1:** Candidate procedures in the Skill Qualification System for portal hypertension

**Endoscopic treatments** Endoscopic injection sclerotherapy for esophageal varices using 5% ethanolamine oleate with contrast iopamidol under fluoroscopy Endoscopic injection sclerotherapy for esophageal varices not under fluoroscopy Endoscopic variceal ligation for esophageal varices and argon plasma coagulation consolidation procedure Endoscopic injection sclerotherapy for gastric varices using cyanoacrylate compound **Interventional radiology** Balloon‐occluded retrograde transvenous obliteration **Surgery** Laparoscopic splenectomy Laparoscopic Hassab's operation (devascularization of the lower esophagus and upper stomach) Open splenectomy Open Hassab's operation Open esophageal transection Open distal splenorenal shunt Other open shunt procedures (left gastric venous caval shunt and inferior mesenteric venous left renal vein shunt)

**TABLE 2:** Examination criteria of endoscopic injection sclerotherapy for esophageal varices using 5% ethanolamine oleate with contrast iopamidol under fluoroscopy

**Common criteria (total 20 points, four points each for criteria 1–5)** Evaluation of treatment completion using photos of endoscopic findings before and at the end of treatment Quickly securing a reliable field of view Cooperation between operator and assistant Smooth and planned technical process and standard procedure time Proper use of the appropriate tools Unsuitable or dangerous techniques (failure) **Specific criteria (total 80 points, five points for each criterion)** Proper endoscopic observation before treatment for esophageal varices Preparation of the tools and equipment Appropriate fluoroscopic field of view Puncture site on the esophageal varices Protrusion length of the puncture needle Needle puncture technique Grasping technique of puncture needle Removal technique of puncture needle Hemostasis technique at the puncture site Comprehensive evaluation of the second and subsequent punctures Confirmation of final hemostasis Immediate determination with 5% ethanolamine oleate with contrast iopamidol injection, intravascular or extravascular Contrast ability of endoscopic varicealography during injection sclerotherapy (EVIS) No shunt outflow or portal vein injection on EVIS; immediate determination and discontinuation of injection, even if so Injection into the left gastric vein and/or short gastric vein on EVIS Inadequate completion of treatment

**TABLE 3:** Examination criteria of balloon‐occluded retrograde transvenous obliteration (B‐RTO)

**Common criteria (total 25 points, five points each for criteria 1–5)** Paying attention to insertion into the heart Immediate recognition to insertion into unrelated veins Field of view captured in the center of monitor and collimated Smooth and planned technical process and standard procedure time Proper use of the appropriate tools Unsuitable or dangerous techniques (failure) **Specific criteria (total 75 points including 10 additional points, five points for each criterion)** Evaluating vascularization of the gastric varices in detail Catheter selection and usage Selection and usage of a guidewire Insertion of the sheath Insertion of a main (B‐RTO) catheter into the left renal vein Insertion of the main (B‐RTO) catheter into the gastro‐renal shunt Insertion of the main catheter or microcatheter into or near the gastric varices Confirmation of other collateral veins by balloon‐occluded retrograde transvenous venography (B‐RTV) Confirmation of gastric varices by B‐RTV Injection method of ethanolamine oleate with contrast iopamidol (EOI) (including injection site) Good accumulation of EOI Appropriate fluoroscopy time Timing of the balloon removal Additional points (Points will be added to the comprehensive evaluation for cases with collateral veins) Catheter insertion into other collateral veins and treatment of the collateral veins (including coil embolization, glucose push technique, etc.) Downgrade technique by inserting a balloon catheter into the gastric varices

**TABLE 4:** Examination criteria of laparoscopic splenectomy

**Common criteria (total 20 points, four points each for criteria 1–5)** Paying attention to organs such as the stomach, spleen, pancreas, and liver Quickly securing a reliable field of view Field of view captured in the center of the monitor Smooth and planned technical process and standard procedure time Proper use of the appropriate tools Unsuitable or dangerous techniques (failure) **Specific criteria (total 80 points, five points for each criterion)** Independence and leadership of the surgeon Cooperation with assistants Port insertion Development of the surgical field Usage of laparoscopic forceps and energy devices Dealing with vessels Hemostasis technique Dealing with the omentum Dealing with the gastrosplenic ligament Dealing with the upper pole of the spleen Dealing with the splenocolic ligament Dealing with the lower pole of the spleen Dissection of the retroperitoneum Dissection of the pancreas tail Dealing with splenic vessels in the splenic hilum Removal of the spleen/Insertion of a drain

### Applicant requirements

To be eligible for accreditation, the applicant must meet the following requirements: 1) be a member of JSPH for 3 years or more, 2) be a board‐certificated doctor in each field, 3) have clinical achievements in treatments for portal hypertension, 4) be able to complete the treatment in the specialized field by oneself, 5) have attended educational seminars of the JSPH in the three fields, 5) be recommended by the local representative of the JSPH, and 6) have sufficient academic achievements for portal hypertension (two journal articles and three presentations at scientific meetings). For example, the applicant for endoscopic treatments must be a board‐certificated doctor of the Japan Gastroenterological Endoscopy Society and have experienced 25 patients or more with esophagogastric varices treated by EIS or endoscopic variceal ligation within 5 years.

Applicants who meet these requirements can then submit a series of documents including an application form, curriculum vitae, list of treated patients, letter of recommendation, a video information form, reference images from a video case, a patient consent form for the video submission, and a list of academic achievements, and an unedited video of an entire procedure. For EIS for esophageal varices using 5% EOI under fluoroscopy, the required reference images from the video case include three or more images of endoscopic varicealography during injection sclerotherapy and two photos of endoscopic findings obtained before and at the end of treatment.

### Application status and accreditation process

From 2014 to 2020, in total, 79 applicants were evaluated by the SQS for portal hypertension. The number of applicants by year was 24, 20, 11, 12, four, three, and five in 2014, 2015, 2016, 2017, 2018, 2019, and 2020, respectively. Among the three specialized fields, the number of applicants was 47 out of 79 (59.5%) in the field of endoscopic treatments, 23 (29.1%) in the field of IVR, and nine (11.4%) in the field of surgery. The most frequent procedures submitted for evaluation in the fields of endoscopic treatments and surgery were EIS for esophageal varices using 5% EOI under fluoroscopy (39/47, 83.0%) and laparoscopic splenectomy (8/9, 88.9%), respectively. In the field of IVR, B‐RTO was the only procedure submitted by the applicants (23/23, 100%).

The chairman of the judging committee anonymously assigned each applicant's video to two judges in the judging committee according to the field of specialization. A grade of greater than 70% for the scoring items assessed by the judges was the criterion of success as above‐mentioned. In cases where the results of the two judges were the same, the decision was made accordingly. When the results of the two judges differed, final judgment was performed by multiple members of the judging committee to obtain consensus. The judges were required to write comments to the applicant describing any inappropriate or dangerous maneuvers shown in the video.

### Statistical analysis

Data were presented as mean ± standard deviation (SD) for continuous variables and percentage for categorical variables. Categorical variables were evaluated using the chi‐square test. As the number of applicants was small between 2018 and 2020, the duration was considered to be one period, and thus, this study was divided into five periods.

Cohen's kappa (κ) coefficient is widely used to measure inter‐rater agreement with binary data. However, it depends strongly on the prevalence of a trait under study and thus can behave paradoxically.^18^, ^19^ Therefore, we used Gwet's AC_1_ coefficient,^18^ which is known to be a more stable agreement coefficient, to evaluate the inter‐rater agreement of success/failure between two judges. To interpret the values of AC_1_, we used the criteria given by Landis and Koch (values <0 as poor, 0.00–0.20 as slight, 0.21–0.40 as fair, 0.41–0.60 as moderate, 0.61–0.80 as substantial, and 0.81–1.00 as almost perfect agreement).^20^ Comparisons of the AC_1_ coefficients between periods, fields, and frequent procedures were conducted by homogeneity score tests.^21^ Inter‐rater reliability of percentages between two judges were investigated by intraclass correlation coefficient (ICC). Comparisons of ICCs between periods, fields, and frequent procedures were conducted by the method based on Fisher's variance stabilizing transformation.^22^ Differences in percentages between three fields or between frequent procedures in the three fields were evaluated using Tukey's multiple comparison test. *P*‐values less than 0.05 were considered to be statistically significant. Statistical analysis was performed with R software (http://cran.r‐project.org).

## RESULTS

The judges observed no unsuitable or dangerous techniques in the 79 videos, and therefore no applicants failed on the basis of this criterion. In 11 of the 79 videos (13.9%), the results of the two judges differed, and the specialized fields of these 11 applicants were endoscopic treatments in three (6.3%), IVR in six (26.1%), and surgery in two (22.2%). The final judgments were performed by the judging committee, and five of the 79 applicants (6.3%) ultimately failed the SQS examination for portal hypertension. These five applicants’ fields were endoscopic treatments in two (4.3%), IVR in two (8.7%), and surgery in one (11.1%). The reasons for the failures were inadequate completion of treatment for esophageal varices and inappropriate puncture technique for gastric varices in the field of endoscopic treatments, video deficiency and inadequate techniques in IVR, and inappropriate laparoscopic techniques, especially hemostasis in surgery. Although the procedures in each field were much different and independent, one applicant passed in both the fields of endoscopic treatments and IVR.

The AC_1_ coefficient for inter‐rater agreement between two judges was 0.84 (95% confidence interval [CI]: 0.74–0.94) and was considered an almost perfect match of two judges. However, the ICC for inter‐rater reliability of percentages was 0.19 (95% CI: –0.003–0.40) and was not statistically significant. The changes in the AC_1_ coefficient and ICC in the five periods are shown in Table 5. These coefficients tended to decrease in 2016 and 2017, but there were no significant differences in the coefficients between the five periods (*p* = 0.626 for AC_1_ and *p* = 0.998 for ICC).

**TABLE 5:** Changes of AC_1_ coefficient and interclass correlation coefficient (ICC) in the five periods

Years	2014	2015	2016	2017	2018–2020
Number of cases	24	20	11	12	12
AC_1_	0.91	0.89	0.78	0.68	0.80
95% CI	0.77–1.00	0.72–1.00	0.40–1.00	0.22–1.00	0.46–1.00
ICC	0.18	0.23	0.16	0.16	0.23
95% CI	–0.23–0.53	–0.22–0.60	–0.44–0.67	–0.41–0.65	–0.32–0.71

Abbreviation: CI, confidence interval.

Means and SDs of the percentages of total points in the three fields (endoscopic treatments, IVR, and surgery) were 87.3 ± 10.3%, 79.4 ± 13.9%, and 80.8 ± 10.2%, respectively. There were statistically significant differences in the percentages between endoscopic treatments and IVR (*p* = 0.0015). Similarly, means and SDs of the percentages for EIS for esophageal varices using 5% EOI under fluoroscopy, B‐RTO, and laparoscopic splenectomy were 88.0 ± 9.4%, 79.4 ± 13.9%, and 80.7 ± 9.8%, respectively, and there were statistically significant differences in the percentages between EIS for esophageal varices using 5% EOI under fluoroscopy and B‐RTO (*p* = 0.0005). The AC_1_ coefficient and ICC in the three fields are shown in Table 6, and these coefficients in the three frequent procedures are shown in Table 7. The ICC in the field of endoscopic treatments was 0.31 (95% CI: 0.03–0.55) and was not well matched but statistically significant. There were no significant differences in the AC_1_ coefficient between the three frequent procedures and in ICC, but there were statistically significant differences in the AC_1_ coefficient between endoscopic treatments and IVR (*p* = 0.021). All judges except for one evaluated no or one failure, but one judge evaluated failure in two of the five applicants for IVR.

**TABLE 6:** AC_1_ coefficient and interclass correlation coefficient (ICC) in the three fields

**Field**	**Endoscopic treatments**	**IVR**	**Surgery**
Number of cases	47	23	9
AC_1_	0.93	0.66	0.72
95% CI	0.85–1.00	0.35–0.98	0.21–1.00
ICC	0.31	–0.13	0.29
95% CI	0.03–0.55	–0.51–0.28	–0.39–0.78

Abbreviations: CI, confidence interval; IVR, interventional radiology.

**TABLE 7:** AC_1_ coefficient and interclass correlation coefficient (ICC) in the three frequent procedures

Procedure	EIS	B‐RTO	LS
Number of cases	39	23	8
AC_1_	0.95	0.66	0.86
95% CI	0.87–1.00	0.35–0.98	0.48–1.00
ICC	0.25	–0.13	0.58
95% CI	–0.07–0.52	–0.51–0.28	–0.09–0.90

Abbreviations: B‐RTO, balloon‐occluded retrograde transvenous obliteration; CI, confidence interval; LS, laparoscopic splenectomy.

EIS: endoscopic injection sclerotherapy for esophageal varices using 5% ethanolamine oleate with contrast iopamidol under fluoroscopy

## DISCUSSION

Although there was almost no statistically significant difference in the inter‐rater reliability based on the percentages of total points using the ICC, the AC_1_ coefficient was 0.84, and the inter‐rater agreement of success/failure between two judges was considered almost perfect in this study. The κ coefficients, which were previously reported in the fields of gastrointestinal and general surgery, and urology on the ESSQS of JSES, were only 0.22–0.59,^1^, ^2^, ^3^, ^4^ and the SQS of JSPH appeared to have a higher inter‐rater agreement. However, these reports on the ESSQS used data between 2004 and 2009,^1^, ^2^, ^3^, ^4^ and the laparoscopic techniques were not necessarily standardized in the early period of laparoscopic surgery since its introduction in 1990.

In Japan, Takase et al. first performed EIS for esophageal varices in 1977.^23^ The EIS technique using 5% EOI under fluoroscopy had been established until 1990.^24^ The Japan Research Society for Sclerotherapy of Esophageal Varices was founded in 1986 and continued to be active in further developing EIS in Japan.^9^ Pertinent issues concerning EIS, such as its indication, prospective technical improvement, and the mechanism underlying sclerosant action underwent continuous studies. Actually, in the 1990s, the techniques using Aethoxysclerol or an overtube not under fluoroscopy were also performed in many Japanese institutions. However, the EIS technique for esophageal varices was almost unified as EIS using 5% EOI under fluoroscopy in the 2000s. The EIS technique has remained uniform and without any added modification for more than 20 years. In contrast, Kanagawa et al. developed B‐RTO in 1990 in Japan.^25^ Chikamori et al. developed a different technique using a different approach route in 1991.^26^ Then, the Cooperative Study Group of B‐RTO was founded in 1998 and has worked to spread safe and effective techniques of B‐RTO in Japan. The techniques of B‐RTO have since been modified repeatedly with regard to balloon catheters, sclerosants, balloon inflation time, and other factors. Therefore, B‐RTO techniques have not been uniform until now. Hashizume et al. first began laparoscopic splenectomy for portal hypertension in 1992,^27^ and the technique had been established until 2000.^28^ Laparoscopic splenectomy has been one of the candidate procedures in the ESSQS of JSES since 2004,^2^, ^3^ and the technique including its associated devices has been almost uniform for more than 10 years.^29^

In the present study, there were statistically significant differences in the scoring percentages between endoscopic treatments and IVR (87.3 ± 10.3% vs. 79.4 ± 13.9%), and also between EIS for esophageal varices using 5% EOI under fluoroscopy and B‐RTO (88.0 ± 9.4% vs. 79.4 ± 13.9%). In addition, the AC_1_ coefficients were 0.93 for endoscopic treatments, 0.66 for IVR, and 0.72 for surgery, and there were significant differences between endoscopic treatments and IVR. The AC_1_ coefficients were 0.95 for EIS for esophageal varices using 5% EOI under fluoroscopy, 0.66 for B‐RTO, and 0.86 for laparoscopic splenectomy. The differences in the percentages and inter‐rater agreement between the judges may be related to the standardization and generalization of the techniques. It is difficult to unify the technique of B‐RTO in its current status. The AC_1_ coefficient was 0.66 for B‐RTO, and while not so low, it was considered necessary to raise the common understanding of the procedure among the IVR judges such as by holding a video clinic.

This study has some limitations. First, the quality of the video submitted between the three fields was much different. The video quality of endoscopic and laparoscopic findings was always clear, but that of B‐RTO fluoroscopy and open surgery was sometimes not good. Second, the sample size was small, especially in the field of surgery. Therefore, 21 judges except for one in the fields of endoscopic treatments and IVR evaluated the unedited videos twice or more, but only three of eight judges in the field of surgery (37.5%) evaluated them twice or more. Third, the number of applicants gradually came down, because most of the specialists for portal hypertension passed the SQS and recently, young doctors who just completed the training have applied it.

In conclusion, the SQS for portal hypertension of the JSPH showed high reliability for video assessments by the judges. This system may contribute to the spread and further development of safe and effective treatments for portal hypertension in Japan.

## CONFLICT OF INTEREST

The authors declare no conflict of interest.

## ETHICS STATEMENT

All procedures performed involving human participants were in accordance with the ethical standards of the institutional and/or national research committee and with the 1964 Helsinki declaration and its later amendments or comparable ethical standards. This study was approved by the Ethics Committee of Oita University Faculty of Medicine (#2073).

## FUNDING INFORMATION

None.

## References

[1] Matsuda T , Ono Y , Terachi T , *et al*. The endoscopic surgical skill qualification system in urological laparoscopy: A novel system in Japan. J Urol 2006; 176: 2168–72. 17070285 10.1016/j.juro.2006.07.034

[2] Mori T , Kimura T , Kitajima M . Skill accreditation system for laparoscopic gastroenterologic surgeons in Japan. Minim Invasive Ther Allied Technol 2010; 19: 18–23. 20095893 10.3109/13645700903492969

[3] Kimura T , Mori T , Konishi F , Kitajima M . Endoscopic surgical skill qualification system in Japan: Five years of experience in the gastrointestinal field. Asian J Endosc Surg 2010; 3: 66–70.

[4] Tanigawa N , Lee Sw , Kimura T , *et al*. The endoscopic surgical skill qualification system for gastric cancer in Japan. Asian J Endosc Surg 2011; 4: 112–5. 22776273 10.1111/j.1758-5910.2011.00082.x

[5] Iwanaka T , Morikawa Y , Yamataka A , *et al*. Skill qualifications in pediatric minimally invasive surgery. Pediatr Surg Int 2011; 27: 727–31. 21365230 10.1007/s00383-011-2871-y

[6] Matsuda T , Kanayama H , Ono Y , *et al*. Reliability of laparoscopic skills assessment on video: 8‐year results of the endoscopic surgical skill qualification system in Japan. J Endourol 2014; 28: 1374–8. 24819163 10.1089/end.2014.0092

[7] Akagi T , Endo H , Inomata M , *et al*.Clinical impact of Endoscopic Surgical Skill Qualification System (ESSQS) by Japan Society for Endoscopic Surgery (JSES) for laparoscopic distal gastrectomy and low anterior resection based on the National Clinical Database (NCD) registry. Ann Gastroenterol Surg 2020; 4: 721–34. 33319163 10.1002/ags3.12384 PMC7726689

[8] Inokuchi K . Current status of surgical treatment of portal hypertension in Japan. Jpn J Surg 1972; 2: 171–85. 4680072 10.1007/BF02470404

[9] Idezuki Y , Japan Research Society for Portal Hypertension,Japanese Research Society for Sclerotherapy of Esophageal Varices . Present status of sclerotherapy and surgical treatment for esophageal varices in Japan. World J Surg 1992; 16: 1193–200. 1455894 10.1007/BF02067101

[10] Japan Research Society for Portal Hypertension . The general rules for recording endoscopic findings on esophageal varices. Jpn J Surg 1980; 10: 84–7. 7373958 10.1007/BF02468653

[11] Inokuchi K , Japan Research Society for Portal Hypertension . Present status of surgical treatment of esophageal varices in Japan: A nationwide survey of 3,588 patients. World J Surg 1985; 9: 171–9. 3872533 10.1007/BF01656275

[12] Idezuki Y , Japan Research Society for Portal Hypertension . General rules for recording endoscopic findings of esophagogastric varices (1991). World J Surg 1995; 19: 420–2. 7638999 10.1007/BF00299178

[13] Inokuchi K , Cooperative Study Group of Portal Hypertension of Japan . Improved survival after prophylactic portal nondecompression surgery for esophageal varices: A randomized clinical trial. Hepatology 1990; 12: 1–6. 2197208 10.1002/hep.1840120102

[14] Hagiwara M , Futagawa S , Suzuki H *et al*. Prophylactic sclerotherapy of high‐risk esophageal varices: Results of a multicentric prospective controlled trial (in Japanese). Portal Hypertension 2001; 7: 140–5.

[15] Tajiri T , Yoshida H , Obara K , *et al*. General rules for recording endoscopic findings of esophagogastric varices (2nd edition). Dig Endosc 2010; 22: 1–9. 20078657 10.1111/j.1443-1661.2009.00929.x

[16] Watanabe N , Toyonaga A , Kojima S , *et al*. Current status of ectopic varices in Japan: Results of a survey by the Japan Society for Portal Hypertension. Hepatol Res 2010; 40: 763–76. 20649816 10.1111/j.1872-034X.2010.00690.x

[17] Kojima S , Watanabe N , Koizumi J , *et al*. Current status of portal vein thrombosis in Japan: Results of a questionnaire survey by the Japan Society for Portal Hypertension. Hepatol Res 2018; 40: 244–54. 10.1111/hepr.12983 28902450

[18] Gwet KLi . Computing inter‐rater reliability and its variance in the presence of high agreement. Br J Math Stat Psychol 2008; 61: 29–48. 18482474 10.1348/000711006X126600

[19] Ohyama T . Statistical inference of agreement coefficient between two raters with binary outcomes. Commun Stat Theory Methods 2020; 49: 2529–39.

[20] Landis JR , Koch GG . The measurement of observer agreement for categorical data. Biometrics 1997; 33: 159–74. 843571

[21] Honda C , Ohyama T . Homogeneity score test of AC_1_ statistics and estimation of common AC_1_ in multiple or stratified inter‐rater agreement studies. BMC Med Res Methodol 2020; 20: 20. 32020851 10.1186/s12874-019-0887-5 PMC7001312

[22] Mian IUH , Shoukri MM . Statistical analysis of interclass correlations from multiple samples with applications to arterial blood pressure data. Stat Med 1997; 16: 1497–514. 9249921 10.1002/(sici)1097-0258(19970715)16:13<1497::aid-sim569>3.0.co;2-7

[23] Takase Y , Ozaki A , Nagoshi K , Okamura T , Iwasaki Y . Injection sclerotherapy of esophageal varices for patients undergoing emergency and elective surgery. Surgery 1982; 92: 474–9. 6981219

[24] Takase Y , Shibuya S , Chikamori F , Orii K , Iwasaki Y . Recurrence factors studied by percutaneous transhepatic portography before and after endoscopic sclerotherapy for esophageal varices. Hepatology 1990; 11: 348–52. 2312049 10.1002/hep.1840110303

[25] Kanagawa H , Mima S , Kouyama H , Gotoh K , Uchida T , Okuda K . Treatment of gastric fundal varices by balloon‐occluded retrograde transvenous obliteration. J Gastroenterol Hepatol 1996; 11: 51–8. 8672742 10.1111/j.1440-1746.1996.tb00010.x

[26] Chikamori F , Shibuya S , Takase Y , Ozaki A , Fukao K . Transjugular retrograde obliteration for gastric varices. Abdom Imaging 1996; 21: 299–303. 8661570 10.1007/s002619900068

[27] Hashizume M , Tomikawa M , Akahoshi T , *et al*. Laparoscopic splenectomy for portal hypertension. Hepatogastroenterology 2002; 49: 847–52. 12064005

[28] Hashizume M , Tanoue K , Akahoshi T , *et al*. Laparoscopic splenectomy: The latest modern technique. Hepatogastroenterology 1999; 46: 820–4. 10370620

[29] Kawanaka H , Akahoshi T , Kinjo N , *et al*. Laparoscopic splenectomy with technical standardization and selection criteria for standard or hand‐assisted approach in 390 patients with liver cirrhosis and portal hypertension. J Am Coll Surg 2015; 221: 354–66. 26206637 10.1016/j.jamcollsurg.2015.04.011

